# The Association of New-Onset Acute Kidney Injury and Mortality in Critically Ill Patients With COVID-19 With Less Severe Clinical Conditions at Admission: A Moderation Analysis

**DOI:** 10.3389/fmed.2022.799298

**Published:** 2022-03-18

**Authors:** Giuseppe Regolisti, Umberto Maggiore, Francesca Di Mario, Micaela Gentile, Giuseppe Daniele Benigno, Ilaria Gandolfini, Valentina Pistolesi, Santo Morabito, Maria Barbagallo, Edoardo Picetti, Enrico Fiaccadori

**Affiliations:** ^1^UOC Clinica e Immunologia Medica, Azienda Ospedaliero-Universitaria di Parma, Dipartimento di Medicina e Chirurgia, Università di Parma, Parma, Italy; ^2^UOC Nefrologia, Azienda Ospedaliero-Universitaria di Parma, Dipartimento di Medicina e Chirurgia, Università di Parma, Parma, Italy; ^3^UOSD Dialisi, Azienda Ospedaliero-Universitaria Policlinico Umberto I, “Sapienza” Università di Roma, Rome, Italy; ^4^UOC Rianimazione 2, Azienda Ospedaliero-Universitaria di Parma, Parma, Italy; ^5^UOC Rianimazione 1, Azienda Ospedaliero-Universitaria di Parma, Parma, Italy

**Keywords:** acute kidney injury, electrolytes, acid–base status, critical illness, COVID-19

## Abstract

Acute kidney injury (AKI), electrolyte, and acid–base disorders complicate the clinical course of critically ill patients with coronavirus-associated disease (COVID-19) and are associated with poor outcomes. It is not known whether the severity of clinical conditions at admission in the intensive care unit (ICU) changes the clinical significance of AKI and/or electrolyte or acid–base disorders developing during ICU stay. We conducted a retrospective study in critically ill patients with COVID-19 to evaluate whether the severity of clinical conditions at admission in the ICU affects the impact of AKI and of serum electrolytes or acid–base status on mortality. We carried out a 28-day retrospective follow-up study on 115 critically ill patients consecutively admitted to ICU for severe COVID-19 at a tertiary care university hospital and surviving longer than 24 h. We collected baseline demographic and clinical characteristics, and longitudinal data on kidney function, kidney replacement therapy, serum electrolytes, and acid–base status. We used Cox proportional hazards multiple regression models to test the interaction between the time-varying variates new-onset AKI or electrolyte or acid–base disorders and Sequential Organ Failure Assessment (SOFA) or Acute Physiology and Chronic Health Evaluation II (APACHE II) score at admission. After adjusting for age, sex, Charlson’s comorbidity index, and AKI present at ICU admission, new-onset AKI was significantly associated with 28-day mortality only in the patients in the lowest and middle SOFA score tertiles [lowest SOFA tertile, hazard ratio (HR) 4.27 (95% CI: 1.27–14.44; *P* = 0.019), middle SOFA tertile, HR 3.17 (95% CI: 1.11–9.04, *P* = 0.031), highest SOFA tertile, HR 0.77 (95% CI: 0.24–2.50; *P* = 0.66); *P* = 0.026 for interaction with SOFA as a continuous variable]. After stratifying for APACHE II tertile, results were similar [adjusted HR (aHR) in the lowest tertile 6.24 (95% CI: 1.85–21.03, *P* = 0.003)]. SOFA or APACHE II at admission did not affect the relationship of serum electrolytes and acid–base status with mortality, except for new-onset acidosis which was associated with increased mortality, with the HR of death increasing with SOFA or APACHE II score (*P* < 0.001 and *P* = 0.013, respectively). Thus, unlike in the most severe critically ill patients admitted to the ICU for COVID-19, in patients with the less severe conditions at admission the development of AKI during the stay is a strong indicator of increased hazard of death.

## Introduction

The incidence of acute kidney injury (AKI) in patients with coronavirus-associated disease discovered in 2019 (COVID-19) has been reported in the recent literature as ranging between 1 and 46% (1), conceivably due to different patient comorbidities (e.g., obesity, diabetes, and hypertension) and especially different clinical setting [i.e., general medicine ward vs. intensive care unit (ICU)]. In the most recently published meta-analysis (2), which included 54 studies with a total of above 30,000 patients, the pooled prevalence of AKI among patients admitted to the ICU was 46%, and 19% of all the ICU patients with COVID-19 were started on kidney replacement therapy (KRT). Out of 5,449 patients with COVID-19 admitted to 13 academic and community hospitals in metropolitan New York with COVID-19 between March 1 and April 5, 2020, almost 90% of the patients needing mechanical ventilation (MV) developed AKI compared with 22% of the patients who were not mechanically ventilated; in those requiring MV, AKI developed within 24 h since intubation (3). Critically ill patients hospitalized with COVID-19 and developing AKI have a poor prognosis, with mortality ranging from 20% to 74% (4–6), and those suffering from KRT-requiring AKI have the worst outcomes (6).

Although AKI at any time during ICU stay was identified as an independent predictor of mortality after adjusting for baseline clinical severity or organ dysfunction at ICU admission (7), to the best of our knowledge no study has examined whether new-onset AKI bears the same prognostic significance regardless of the degree of severity or multiorgan dysfunction at the time of ICU admission.

Besides AKI, electrolyte (8–12) and acid–base (13, 14) derangements are common in patients admitted for severe COVID-19, with hyponatremia (15, 16) and acidemia (14) especially bearing a negative prognostic impact. However, information is lacking regarding how baseline clinical severity or organ dysfunction at ICU admission affects the impact on mortality of new-onset electrolyte or acid–base disorders.

Thus, we aimed at exploring whether the degree of organ dysfunction at ICU admission modifies the impact of new-onset AKI and of electrolyte or acid–base derangements on the risk of death in critically ill patients with COVID-19.

## Materials and Methods

### Patients

We conducted an observational retrospective study in all the patients consecutively admitted to the medical and surgical ICUs of a tertiary care University hospital (Unità Operativa di Rianimazione 1 e Unità Operativa di Rianimazione 2, Azienda Ospedaliero-Universitaria di Parma, Parma, Italy) for COVID-19-related interstitial pneumonia and severe respiratory failure from February 23rd to May 1st, 2020.

The infection with severe acute respiratory syndrome coronavirus 2 (SARS-CoV-2) was confirmed by the detection of SARS-CoV-2 RNA by reverse transcription-polymerase chain reaction (RT-PCR) on nasopharyngeal swab specimens, while the presence and the extension of interstitial pneumonia were assessed by high-resolution chest tomography. Anonymized demographical, clinical, and laboratory data, and information on therapy were extracted from the electronic records and physical charts of the patients.

### Data Collection

We collected demographic (age and sex), clinical (date of symptom onset, time interval from symptom onset to ICU admission), comorbidities [arterial hypertension (HTN), diabetes (DM), obesity, coronary artery disease (CAD), chronic kidney disease (CKD), chronic obstructive pulmonary disease (COPD), sepsis, and cancer], need for vasoactive amines, need for MV, the Acute Physiology And Chronic Health Evaluation II (APACHE II) and Sequential Organ Failure Assessment (SOFA) scores, and laboratory data [complete blood count and differential, serum values of glucose, urea, creatinine, sodium, potassium, calcium, phosphate, liver enzymes, total and direct bilirubin, creatine phosphokinase (CPK), C-reactive protein (CRP), and procalcitonin (PCT)] at the time of ICU admission. We also collected vital signs (blood pressure, heart rate, respiratory rate, and body temperature), the need for MV, and blood gas analysis data at the time of ICU admission, as well as at 7, 14, 21, and 28 days since ICU admission and/or at the time of discharge from the ICU. All patients had an arterial line inserted at the time of ICU admission, which was left in place during the whole ICU stay. Serum values of urea, creatinine, sodium, and potassium were collected daily.

The study was approved by the institutional Ethics Committee (Comitato Etico dell’Area Vasta Emilia Nord, approval no. 1264/2020/OSS*/AOUPR issued on January 12, 2021).

### Definitions

We assessed AKI and its severity based on the change in serum creatinine value vs. value at baseline, according to the Kidney Disease Improving Global Outcomes (KDIGO) definitions (17). When available, we took the last serum creatinine value within 7–365 days prior to the hospital admission as baseline serum creatinine; alternatively, we considered serum creatinine at the time of hospital admission as the baseline value.

We defined hyponatremia as a serum sodium value <136 mmol/L, and hypernatremia as a serum sodium value >144 mmol/L; we defined hypokalemia as a serum potassium value <3.6 mmol/L, and hyperkalemia as a serum potassium value >5.0 mmol/L. We defined metabolic acidosis as an arterial plasma bicarbonate value <22 mmol/L, and metabolic alkalosis as an arterial plasma bicarbonate value >28 mmol/L.

We defined sepsis according to the Third International Consensus Definitions for Sepsis and Septic Shock (Sepsis-3) (18).

We defined CKD based on the presence of abnormalities of kidney function or structure for longer than 3 months, according to the 2012 KDIGO guidelines (19). As we did not have data on proteinuria, microscopic hematuria, or other abnormalities in urine sediment, previous CKD was established based on either estimated glomerular filtration rate (eGFR) derived from serum creatinine values by the CKD-epidemiology collaboration (EPI) equation, in patients with at least two values of serum creatinine available within at least 90 days prior to hospital admission, or on CKD diagnosis reported in medical charts in patients without available previous serum creatinine values.

The study endpoint was death within 28 days of follow-up.

### Data Analysis

All analyses were performed with Stata SE release 17.0 (2021, StataCorp LLC, College Station, TX, United States). We regarded two-tailed *P*-values of less than 0.05 as statistically significant unless otherwise stated.

We carried out two-sample comparisons of continuous variables with the Mann–Whitney test, and categorical variables with the Fisher’s exact test. To enhance the insight into, and ease the interpretation of the data findings, we divided patients according to SOFA tertiles at admission. The number of patients in each tertile category was not even because of tied values in the SOFA score. We tested the trend of the baseline variables across the SOFA tertiles using the Cuzick rank test for continuous variables, the Jonckheere–Terpstra test for ordered variables, and the exact Cochran–Armitage test for dichotomous variables.

Our primary hypothesis was that, in critically ill patients with COVID-19, AKI may have born a heavier prognostic impact in the patients with less severe organ dysfunction at the time of ICU admission. In fact, AKI may herald an acute deterioration of clinical conditions in otherwise stable patients, whereas in patients with multi-organ dysfunction the subsequent clinical evolution is already captured by general scores of clinical severity. Thus, we performed the analyses on the moderator effect of baseline SOFA or APACHE II scores by fitting separate multivariable Cox proportional hazards regression models that included new-onset AKI, hyponatremia, hypernatremia, hypokalemia, hyperkalemia, metabolic alkalosis, and acidosis as time-varying indicator variates; in each model, we fitted an interaction term between the indicator variable (e.g., new-onset AKI) and baseline SOFA or APACHE II score fitted as a continuous linear variable. If the interaction term was statistically significant, we regarded the moderator effect as being present. We finally displayed the hazard ratio (HR) estimated within each tertile of baseline SOFA and APACHE II score in order to generate a plot that is easy to interpret. In order to generate such a plot, we fitted multivariable Cox proportional hazards regression models in which SOFA or APACHE II score was included as indicator variables for tertiles as opposed to the numerical value. We tested the linearity of continuous variables with fractional polynomials, and the proportional hazards assumption on the basis of Schoenfeld residuals.

In order to visually appraise the timing of the change in the hazard of death and AKI since ICU admission, we plotted the cumulative hazard of death along with the cumulative hazard of AKI.

## Results

### Patients

**FIGURE 1:**
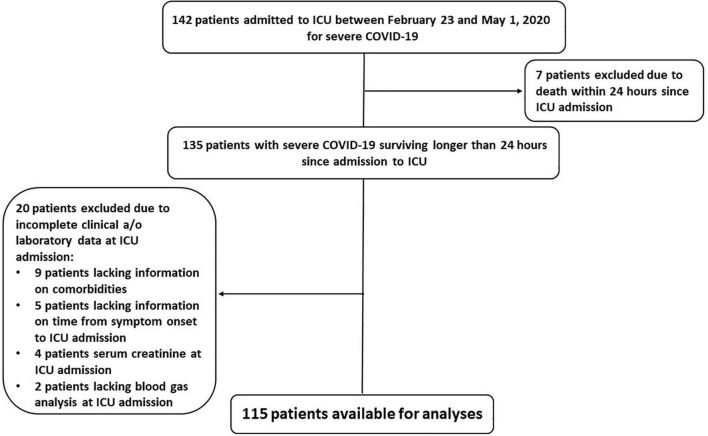
Flow chart of the process of selection of the study population.

From February 23 to May 1, 2020, we enrolled 142 consecutive patients with SARS-CoV-2 infection confirmed by molecular analysis on nasopharyngeal swab specimens and interstitial pneumonia with severe respiratory failure requiring ICU admission and ventilatory support. We excluded seven patients who died within 24 h since ICU admission; we excluded twenty more patients lacking complete data at ICU admission. Therefore, we conducted our analyses on 115 patients (Figure 1).

The mean patient age was 59.7 years (SD 8.9 years), and most (82%) were males. Arterial hypertension (48%) was the most prevalent comorbidity in this population; diabetes and obesity were present in 13% and 15% of the patients, respectively. One-fifth of the patients had diagnostic criteria of sepsis at ICU admission. Mean time interval between symptom onset and ICU admission was 13 (SD 8) days. The main symptoms at hospital admission were fever (92%) and dyspnea (61%) (Table 1).

**TABLE 1:** Demographical and clinical data of patients according to tertiles of SOFA score at ICU admission.

		SOFA tertile									*P*-value*		
	All patients			Lower tertile			Middle tertile			Upper tertile			
**Number of patients**	**115**			**49**			**29**			**37**			
Age, years	**115**	59.7	(8.9)	**49**	57.3	(9.7)	**29**	62.2	(6.8)	**37**	60.8	(8.8)	0.163
Sex	**115**			**49**			**29**			**37**			0.075
Males		21	18.3%		12	24.5%		6	20.7%		3	8.1%	
Females		94	81.7%		37	75.5%		23	79.3%		34	91.9%	
APACHE II score	**115**	18.7	(6.8)	**49**	13.8	(3.4)	**29**	20.4	(5.7)	**37**	24.0	(6.3)	0.000
SOFA score	**115**	6.2	(2.6)	**49**	3.8	(1.0)	**29**	6.6	(0.5)	**37**	9.2	(1.5)	0.000
Charlson’s score – Age	**115**	2.0	(1.2)	**49**	1.8	(1.1)	**29**	2.3	(1.2)	**37**	2.1	(1.3)	0.198
Obesity	**115**			**49**			**29**			**37**			0.922
No		98	85.2%		43	87.8%		23	79.3%		32	86.5%	
Yes		17	14.8%		6	12.2%		6	20.7%		5	13.5%	
Hypertension	**115**			**49**			**29**			**37**			0.790
No		60	52.2%		26	53.1%		16	55.2%		18	48.6%	
Yes		55	47.8%		23	46.9%		13	44.8%		19	51.4%	
Diabetes	**115**			**49**			**29**			**37**			0.327
No		100	87.0%		44	89.8%		26	89.7%		30	81.1%	
Yes		15	13.0%		5	10.2%		3	10.3%		7	18.9%	
CKD	**115**			**49**			**29**			**37**			1.000
No		109	94.8%		48	98.0%		24	82.8%		37	100.0%	
Yes		6	5.2%		1	2.0%		5	17.2%		0	0.0%	
CAD	**115**			**49**			**29**			**37**			0.440
No		108	93.9%		44	89.8%		29	100.0%		35	94.6%	
Yes		7	6.1%		5	10.2%		0	0.0%		2	5.4%	
COPD	**115**			**49**			**29**			**37**			0.601
No		111	96.5%		48	98.0%		28	96.6%		35	94.6%	
Yes		4	3.5%		1	2.0%		1	3.4%		2	5.4%	
Cancer	**115**			**49**			**29**			**37**			1.000
No		110	95.7%		47	95.9%		27	93.1%		36	97.3%	
Yes		5	4.3%		2	4.1%		2	6.9%		1	2.7%	
Sepsis	**115**			**49**			**29**			**37**			0.016
No		92	80.0%		45	91.8%		21	72.4%		26	70.3%	
Yes		23	20.0%		4	8.2%		8	27.6%		11	29.7%	
MBP, mmHg	**114**	79.5	(11.6)	**49**	82.3	(12.1)	**29**	80.1	(11.4)	**36**	75.1	(9.9)	0.006
Heart rate, beats/min	**114**	83.2	(20.3)	**49**	82.4	(15.2)	**29**	81.2	(23.2)	**36**	85.8	(23.9)	0.893
Respiratory rate, breaths/min	**115**	20.0	(4.5)	**49**	20.9	(5.3)	**29**	19.4	(3.8)	**37**	19.1	(3.6)	0.046
Body temperature,°C	**114**	36.7	(1.0)	**49**	36.6	(0.9)	**29**	36.7	(1.1)	**36**	36.7	(1.2)	0.873
Fever	**115**			**49**			**29**			**37**			0.167
No		9	7.8%		3	6.1%		0	0.0%		6	16.2%	
Yes		106	92.2%		46	93.9%		29	100.0%		31	83.8%	
Dyspnea	**115**			**49**			**29**			**37**			0.193
No		45	39.1%		19	38.8%		18	62.1%		8	21.6%	
Yes		70	60.9%		30	61.2%		11	37.9%		29	78.4%	
Cough	**115**			**49**			**29**			**37**			0.616
No		59	51.3%		29	59.2%		10	34.5%		20	54.1%	
Yes		56	48.7%		20	40.8%		19	65.5%		17	45.9%	
Diarrhea	**115**			**49**			**29**			**37**			0.893
No		107	93.0%		47	95.9%		25	86.2%		35	94.6%	
Yes		8	7.0%		2	4.1%		4	13.8%		2	5.4%	
Oliguria	**48**			**10**			**13**			**25**			0.142
No		40	83.3%		9	90.0%		13	100.0%		18	72.0%	
Yes		8	16.7%		1	10.0%		0	0.0%		7	28.0%	
Time from symptom onset to ICU admission, days	**114**	12.6	(7.7)	**49**	14.3	(7.1)	**29**	12.6	(6.8)	**36**	10.3	(8.7)	0.014
Fluid balance in the first 24 h, mL	**110**	1364.0	(1245.5)	**47**	1211.4	(1189.2)	**28**	1053.1	(914.9)	**35**	1817.7	(1439.3)	0.081
Vasopressors	**115**			**49**			**29**			**37**			
No		64	55.7%		47	95.9%		12	41.4%		5	13.5%	0.000
Yes		51	44.3%		2	4.1%		17	58.6%		32	86.5%	
MV	**115**						**29**			**37**			
No		13	11.3%		12	24.5%		1	3.4%		0	0.0%	0.000
Yes		102	88.7%		37	75.5%		28	96.6%		37	100.0%	
Treatment with hydroxychloroquine	**115**			**49**			**29**			**37**			0.561
No		8	7.0%		3	6.1%		1	3.4%		4	10.8%	
Yes		107	93.0%		46	93.9%		28	96.6%		33	89.2%	
Treatment with antiviral drugs	**115**			**49**			**29**			**37**			0.067
No		4	3.5%		4	8.2%		0	0.0%		0	0.0%	
Yes		111	96.5%		45	91.8%		29	100.0%		37	100.0%	
Treatment with azithromycin	**115**			**49**			**29**			**37**			0.063
No		30	26.1%		9	18.4%		7	24.1%		14	37.8%	
Yes		85	73.9%		40	81.6%		22	75.9%		23	62.2%	
Treatment with tocilizumab	**115**			**49**			**29**			**37**			0.013
No		101	87.8%		39	79.6%		26	89.7%		36	97.3%	
Yes		14	12.2%		10	20.4%		3	10.3%		1	2.7%	
Treatment with colchicine	**115**			**49**			**29**			**37**			1.000
No		107	93.0%		46	93.9%		26	89.7%		35	94.6%	
Yes		8	7.0%		3	6.1%		3	10.3%		2	5.4%	

Data are expressed as mean (SD), or count and percentage. *P-values refer to tests for trend (Cuzick rank test for continuous variables, Jonckheere-Terpstra test for ordered variables, and exact Cochran-Armitage test for dichotomous variables). APACHE II, Acute Physiology Assessment and Chronic Health Evaluation II; CAD, coronary artery disease; CKD, chronic kidney disease; COPD, chronic obstructive pulmonary disease; ICU, intensive care unit; MBP, mean blood pressure; MV, mechanical ventilation; SOFA, Sequential Organ Failure Assessment. The bold values indicate that the number of patients for the total population and each tertile of SOFA score that were available for statistical analyses for each variable.

The patients had severely compromised clinical conditions at ICU admission, as shown by a mean 6.2 (SD 2.6) SOFA score. Due to a rapid worsening in respiratory failure and hemodynamic instability, 89% of the patients required MV, and 44% required vasoactive amines during ICU stay.

### Acute Kidney Injury Incidence and Patient Characteristics

A total of eight (7.0%) patients had AKI at ICU admission, based on serum creatinine change with respect to the first value obtained at the admission to the Emergency Room. Thirty-four patients (29.6%) developed new-onset AKI during ICU stay; of these, 22 patients (64.7%) developed stage 1 AKI, 4 patients (11.8%) developed stage 2 AKI, and 8 patients (23.5%) developed stage 3 AKI. Data for true baseline serum creatinine values, as collected 7–365 days prior to hospital admission, were available for a total of 63 patients (54.8%). There were no significant differences in true baseline serum creatinine values between the patients who had and those who had not AKI at the time of ICU admission (0.93 (SD 0.14) vs. 0.91 (SD 0.17) mg/dL, respectively, *P* = 0.79), nor between those who did and those who did not develop new-onset AKI during ICU stay (0.92 (SD 0.10) vs. 0.91 (SD 0.19) mg/dL, respectively, *P* = 0.90).

**FIGURE 2:**
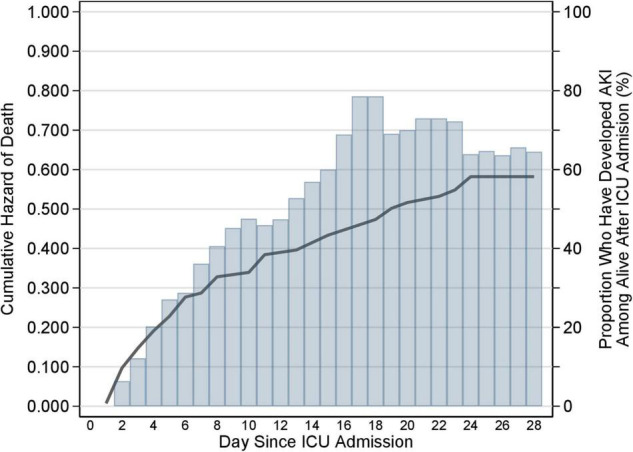
Cumulative hazard of death and cumulative incidence of AKI during ICU stay. Cumulative hazard of death (solid line) and cumulative incidence of AKI (histogram) during the 28-day follow-up in the ICU. The hazard of death and the incidence of AKI is the daily risk of dying and developing AKI, respectively. The graph represents the cumulative sum of each of the two rates. If the cumulative hazard of death is constant over time in the ICU, then the cumulative hazard will rise linearly with days since admission in the ICU (*x*-axis); if the hazard increases with time in the ICU, the cumulative hazard will rise non-linearly showing an increase in slope with increasing days since admission; if the hazard decreases with time in the ICU the cumulative hazard will still rise, but now with a decrease in the slope. The same applies to the cumulative incidence of AKI. The graph shows that both the hazard of death and the incidence of AKI tended to decrease after the first 8 days after ICU admission. AKI, acute kidney injury; ICU, intensive care unit.

Both the hazard of death and the incidence of AKI tended to decrease after the first 8 days after ICU admission (Figure 2).

Mean age and prevalence of comorbidities were similar between the patients with and those without new-onset AKI (Supplementary Table 1). The prevalence of sepsis was numerically higher in the former than in the latter, although this difference was not statistically significant (29.4 vs. 16.0%, *P* = 0.13) (Supplementary Table 1). Mean blood pressure at the time of ICU admission was marginally lower in the patients who did compared with those who did not develop new-onset AKI (76.5 (SD 10.0) vs. 80.7 (SD 12.0) mmHg, *P* = 0.056), and a higher fraction of patients with new-onset AKI needed vasoactive support with amines at some time during ICU stay (62.9% vs. 36.1% in the patients who did not develop AKI; *P* = 0.007). The need for MV was not significantly different between the patients who did and those who did not develop AKI (97.1% vs. 85.2%, *P* = 0.10). Except for a lower frequency of treatment with azithromycin in the patients with new-onset AKI, we did not observe significant differences in the frequency of other drug treatments with putative efficacy on SARS-CoV-2 infection at the time of this study (Supplementary Table 1).

Compared with the patients who did not, those who developed new-onset AKI had higher serum values of creatinine, urea, sodium, and CRP, while they had lower values of serum calcium, arterial blood PH, and plasma bicarbonate, at the time of ICU admission (Supplementary Table 1).

The mean SOFA score was significantly higher in the patients who did than in those who did not develop new-onset AKI [7.2 (SD 2.4) vs. 5.8 (SD 2.6), *P* = 0.004], whereas the mean APACHE II score was not significantly different in the two groups [19.1 (SD 6.8) vs. 18.6 (SD 6.8), respectively, *P* = 0.70] (Supplementary Table 1).

### Differences in Demographic, Clinical, and Laboratory Data Across Tertiles of Sequential Organ Failure Assessment Score at Intensive Care Unit Admission

Mean age and prevalence of comorbidities were not significantly different among all patients as partitioned into tertiles of SOFA score. As shown in Table 1, the characteristics that showed a significant trend across SOFA tertiles were mean blood pressure values (*P* = 0.006), need of vasopressors for hemodynamic support (*P* < 0.001), need for MV (*P* < 0.001), the time from symptom onset to ICU admission (*P* = 0.014), and treatment with tocilizumab (*P* = 0.013).

We also observed an increasing trend in the prevalence of sepsis (*P* = 0.011), which reached nearly 30% in the highest tertile (Table 1); accordingly, as shown in Table 2, there was an increasing trend of serum CRP (*P* = 0.035) and serum procalcitonin (0.012) at the time of ICU admission across SOFA tertiles. Finally, we detected an increasing trend of serum creatinine (*P* < 0.001) and urea (*P* = 0.009) concentration at the time of ICU admission (Table 2). In contrast, with the possible exception of serum calcium (*P* = 0.053), serum electrolyte concentrations did not change significantly across SOFA tertiles (Table 2).

**TABLE 2:** Laboratory data of patients according to tertiles of SOFA score at ICU admission.

		SOFA tertile											
	All patients			Lower tertile			Middle tertile			Upper tertile			*P*-value*
**Number of patients**	**115**			**49**			**29**			**37**			
WBC count, ×103/μL	**115**	10735.3	(5518.2)	**49**	9897.2	(4600.9)	**29**	10903.8	(6394.6)	**37**	11713.0	(5868.5)	0.195
Hemoglobin, g/dL	**115**	12.5	(1.7)	**49**	12.6	(1.5)	**29**	12.3	(1.9)	**37**	12.4	(1.8)	0.674
MCV, fL	**115**	89.4	(12.3)	**49**	88.3	(12.5)	**29**	90.3	(7.3)	**37**	90.2	(15.0)	0.048
PLT count, ×103/μL	**115**	260217.4	(100229.5)	**49**	285469.4	(91974.2)	**29**	241758.6	(87285.9)	**37**	241243.2	(114433.5)	0.007
Serum glucose, mg/dL	**106**	156.7	(61.1)	**45**	143.4	(50.5)	**26**	161.1	(57.1)	**35**	170.4	(73.3)	0.026
Serum urea, mg/dL	**113**	56.3	(45.6)	**48**	44.2	(15.5)	**28**	54.5	(39.9)	**37**	73.5	(66.8)	0.009
Serum creatinine, mg/dL	**115**	0.9	(0.7)	**49**	0.7	(0.2)	**29**	0.9	(0.4)	**37**	1.3	(1.1)	0.000
Serum sodium, mmol/L	**115**	137.5	(4.0)	**49**	136.9	(3.2)	**29**	137.9	(4.2)	**37**	137.9	(4.7)	0.407
Serum potassium, mmol/L	**115**	4.0	(0.5)	**49**	3.9	(0.5)	**29**	3.9	(0.6)	**37**	4.1	(0.6)	0.108
Serum chloride, mmol/L	**112**	101.5	(4.6)	**48**	100.8	(3.5)	**27**	101.6	(5.3)	**37**	102.5	(5.2)	0.040
Serum calcium, mg/dL	**94**	8.0	(0.7)	**40**	8.2	(0.6)	**22**	7.8	(0.9)	**32**	7.9	(0.5)	0.053
Serum total bilirubin, mg/dL	**107**	0.9	(0.5)	**46**	0.8	(0.3)	**26**	1.1	(0.8)	**35**	0.9	(0.5)	0.689
AST, UI/L	**109**	79.7	(99.5)	**47**	76.3	(75.5)	**26**	66.7	(47.7)	**36**	93.4	(145.2)	0.461
ALT, UI/L	**107**	67.0	(75.6)	**47**	72.9	(79.4)	**25**	59.3	(94.3)	**35**	64.5	(54.2)	0.852
LDH, UI/L	**96**	602.7	(218.3)	**43**	590.4	(204.2)	**24**	595.3	(218.1)	**29**	627.1	(243.2)	0.785
CPK, UI/L	**96**	313.3	(820.4)	**41**	228.2	(294.8)	**24**	242.6	(247.2)	**31**	480.7	(1387.8)	0.229
INR	**109**	1.4	(0.3)	**45**	1.5	(0.3)	**29**	1.4	(0.3)	**35**	1.4	(0.3)	0.053
aPTT ratio	**111**	1.0	(0.3)	**46**	1.0	(0.2)	**29**	1.0	(0.3)	**36**	1.0	(0.3)	0.674
D-Dimer, ng/mL	**102**	4384.1	(3395.6)	**47**	4169.5	(3242.4)	**25**	4679.4	(3353.3)	**30**	4474.3	(3743.9)	0.949
Serum CRP, mg/L	**88**	160.0	(81.7)	**43**	154.9	(79.0)	**20**	122.5	(82.9)	**25**	198.7	(71.0)	0.035
Serum PCT, ng/mL	**113**	4.2	(30.3)	**49**	1.2	(4.0)	**29**	1.0	(1.5)	**35**	11.0	(54.2)	0.012
Serum troponine, ng/L	**98**	43.9	(76.1)	**39**	22.5	(43.3)	**24**	62.2	(112.8)	**35**	55.3	(70.1)	0.000
Arterial blood pH	**115**	7.4	(0.1)	**49**	7.4	(0.1)	**29**	7.4	(0.1)	**37**	7.3	(0.1)	0.005
Partial pressure of O_2_ in arterial blood, mmHg	**115**	80.2	(29.5)	**49**	76.5	(28.2)	**29**	85.0	(27.8)	**37**	81.4	(32.5)	0.466
Partial pressure of CO_2_ in arterial blood, mmHg	**115**	46.5	(11.1)	**49**	44.2	(9.0)	**29**	47.4	(13.5)	**37**	48.9	(11.2)	0.036
Bicarbonate concentration in arterial blood, mmol/L	**115**	24.5	(4.0)	**49**	25.1	(3.0)	**29**	24.7	(4.4)	**37**	23.4	(4.5)	0.006
eGFR, mL/min/1.73 m^2^	**56**	85.6	(13.1)	**26**	88.5	(12.0)	**17**	82.2	(15.8)	**13**	84.2	(10.6)	0.261
Hypernatremia	**115**			**49**			**29**			**37**			0.120
No		110	95.7%		49	100.0%		27	93.1%		34	91.9%	
Yes		5	4.3%		0	0.0%		2	6.9%		3	8.1%	
Hyponatremia	**115**			**49**			**29**			**37**			0.431
No		94	81.7%		42	85.7%		23	79.3%		29	78.4%	
Yes		21	18.3%		7	14.3%		6	20.7%		8	21.6%	
Hyperkalemia	**115**			**49**			**29**			**37**			1.000
No		114	99.1%		49	100.0%		28	96.6%		37	100.0%	
Yes		1	0.9%		0	0.0%		1	3.4%		0	0.0%	
Hypokalemia	**115**			**49**			**29**			**37**			0.279
No		97	84.3%		40	81.6%		23	79.3%		34	91.9%	
Yes		18	15.7%		9	18.4%		6	20.7%		3	8.1%	
Metabolic alkalosis	**115**			**49**			**29**			**37**			0.650
No		95	82.6%		41	83.7%		25	86.2%		29	78.4%	
Yes		20	17.4%		8	16.3%		4	13.8%		8	21.6%	
Metabolic acidosis	**115**			**49**			**29**			**37**			0.104
No		89	77.4%		40	81.6%		25	86.2%		24	64.9%	
Yes		26	22.6%		9	18.4%		4	13.8%		13	35.1%	

Data are expressed as mean (SD), or count and percentage. *P-values refer to tests for trend (Cuzick rank test for continuous variables, Jonckheere–Terpstra test for ordered variables, and exact Cochran–Armitage test for dichotomous variables). ALT, alanine aminotransferase; aPTT, activated partial thromboplastin time; AST, aspartate aminotransferase; CPK, creatine phosphokinase; eGFR, estimated glomerular filtration rate; CRP, C-reactive protein; ICU, intensive care unit; INR, international normalized ratio; MCV, mean corpuscular volume; PCT, procalcitonin; PLT, platelet; SOFA, Sequential Organ Failure Assessment; WBC, white blood cell. The bold values indicate that the number of patients for the total population and each tertile of SOFA score that were available for statistical analyses for each variable.

The frequency of AKI both at hospital admission and at ICU admission rose progressively from the lowest to the highest SOFA tertile (*P* = 0.017 and *P* = 0.013, respectively). The frequency of new-onset AKI during the ICU stay also increased progressively (*P* = 0.001) across tertiles of SOFA score (Table 3). Although the majority of patients developed stage 1 AKI, the incidence of stage 3 AKI increased from 4.1% in the lowest to 13.5% in the highest tertile (Table 3). The ranges of the continuous variables shown in Tables 1–3 are reported in Supplementary Table 2.

**TABLE 3:** Event data of patients according to tertiles of SOFA score at ICU admission.

		SOFA tertile											
	All patients			Lower tertile			Middle tertile			Upper tertile			*P*-value*
**Number of patients**	**115**			**49**			**29**			**37**			
AKI at hospital admission	**115**			**49**			**29**			**37**			0.029
No		98	85.2%		46	93.9%		24	82.8%		28	75.7%	
Yes		17	14.8%		3	6.1%		5	17.2%		9	24.3%	
AKI at ICU admission	**115**			**49**			**29**			**37**			0.024
No		107	93.0%		48	98.0%		28	96.6%		31	83.8%	
Yes		8	7.0%		1	2.0%		1	3.4%		6	16.2%	
AKI developing in the ICU	**115**			**49**			**29**			**37**			0.001
No		81	70.4%		41	83.7%		22	75.9%		18	48.6%	
Yes		34	29.6%		8	16.3%		7	24.1%		19	51.4%	
AKI stage	**115**			**49**			**29**			**37**			0.001
No AKI		81	70.4%		41	83.7%		22	75.9%		18	48.6%	
Stage 1 AKI		22	19.1%		5	10.2%		5	17.2%		12	32.4%	
Stage 2 AKI		4	3.5%		1	2.0%		1	3.4%		2	5.4%	
Stage 3 AKI		8	7.0%		2	4.1%		1	3.4%		5	13.5%	
Days with NIV or MV	**115**	28.4	(27.2)	**49**	30.5	(25.1)	**29**	26.4	(30.2)	**37**	27.1	(27.9)	0.418
Length of ICU stay, days	**115**	30.4	(29.8)	**49**	33.3	(28.1)	**29**	28.6	(32.7)	**37**	28.0	(30.3)	0.221
Death at 28 days since ICU admission	**115**			**49**			**29**			**37**			0.163
No		69	60.0%		35	71.4%		13	44.8%		21	56.8%	
Yes		46	40.0%		14	28.6%		16	55.2%		16	43.2%	

Data are expressed as mean (SD), or count and percentage. *P-values refer to tests for trend (Cuzick rank test for continuous variables, Jonckheere–Terpstra test for ordered variables, and exact Cochran–Armitage test for dichotomous variables). AKI, acute kidney injury; ICU, intensive care unit; MV, mechanical ventilation; NIV, non-invasive ventilation; SOFA, Sequential Organ Failure Assessment. The bold values indicate that the number of patients for the total population and each tertile of SOFA score that were available for statistical analyses for each variable.

Due to a rapid deterioration in the clinical conditions of most of the patients with AKI, with worsening respiratory failure notwithstanding MV, severe hemodynamic instability, and multiorgan failure, KRT was considered in only 4 out of 8 (50.0%) patients with stage 3 AKI. These four patients were treated with sustained low-efficiency dialysis (SLED); a detailed analysis of these treatments has been published previously (20).

Although the percentage of patients who died by 28-day of follow-up increased from 28.6% in the lowest to 55.2% in the middle and was 43.2% in the highest SOFA tertile, this trend did not reach statistical significance (*P* = 0.13).

### Association Between New-Onset Acute Kidney Injury and Death

**FIGURE 3:**
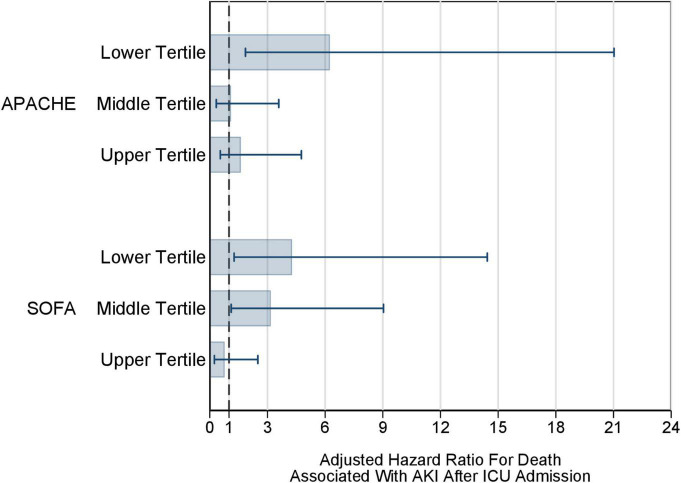
Multivariable adjusted AKI-associated risk of death in the ICU across tertiles of SOFA or APACHE II score. Multivariable-adjusted hazard ratio of death associated with AKI during ICU stay, stratified for each tertile of the SOFA (lower part) or APACHE II (upper part) score at ICU admission. The shaded bars represent the adjusted hazard ratio, the horizontal lines represent 95% CI. Horizontal lines crossing the dotted vertical line indicate that the hazard ratio was not statistically significant. AKI, acute kidney injury; APACHE II, Acute Physiology and Chronic Health Evaluation II; ICU, intensive care unit; SOFA, Sequential Organ Failure Assessment.

After adjusting for age, sex, Charlson’s comorbidity index, AKI present at ICU admission, and SOFA score, in the overall study population the association of new-onset AKI during ICU stay with mortality at 28 days was only of borderline statistical significance [adjusted HR (aHR): 1.90; 95% CI: 0.96–3.75; *P* = 0.066] (Supplementary Table 3). However, there was evidence of a moderator effect, with the strength of the association between AKI during ICU stay and death depending on the level of the baseline SOFA score, i.e., the higher the SOFA score the lower the strength of the association between AKI during ICU stay and death. In fact, at the mean level of SOFA, the aHR was 2.66 (95% CI: 1.30–5.43; *P* = 0.007); for every unit increase in SOFA the aHR decreased by 0.72 times (95% CI: 0.54–0.96; *P* = 0.026). In order to ease the interpretation of the findings on the moderator effect of SOFA score, we divided SOFA score into tertiles and estimated the aHR within each SOFA tertile. In the lowest SOFA tertile aHR was 4.27 (95% CI: 1.27–14.44; *P* = 0.019), in the middle SOFA tertile aHR was 3.17 (95% CI: 1.11–9.04, *P* = 0.031), and in the highest SOFA tertile aHR was 0.77 (95% CI: 0.24–2.50; *P* = 0.66) (Figure 3, lower part). After stratifying for APACHE II tertile, results were similar [aHR in the lowest tertile: 6.24 (95% CI 1.85–21.03), *P* = 0.003] (Figure 3, upper part). In fact, after adjusting for age, sex, Charlson’s comorbidity index, AKI present at ICU admission, and APACHE II score, in the overall study population the association of new-onset AKI during ICU stay with mortality at 28 days was statistically significant [aHR: 2.15; 95% CI: 1.10–4.20; *P* = 0.025] (Supplementary Table 3).

### Incidence of Electrolyte and Acid–Base Disorders and Their Association With Death

The incidence of hyponatremia was 18.3% in the overall population, while hypernatremia was infrequent (4.3%). Similarly, hypokalemia developed in 15.7% of the patients during ICU stay, while hyperkalemia was rarely observed (0.9%). The incidence of metabolic acidosis and metabolic alkalosis was 22.6 and 17.4%, respectively (Table 2).

After adjusting for age, sex, Charlson’s comorbidity index, AKI present at ICU admission, and SOFA or APACHE II score none among hypo-hypernatremia, hypo-hyperkalemia developing during ICU stay were associated with mortality (data not shown). The same held true for metabolic alkalosis, while there was a strong and significant interaction between metabolic acidosis and SOFA or APACHE II score, with the HR of death associated with metabolic acidosis increasing by 1.75 times [(95% CI 1.34–2.29), *P* < 0.001] for each unit of SOFA score and by 1.19 times [(95% CI 1.04–1.37), *P* = 0.013] for each unit of APACHE II score. However, most cases of metabolic acidosis occurred in the most severe patients based on SOFA score at admission (Table 2).

## Discussion

The main finding of this study is that new-onset AKI developing in the ICU in critically ill patients with COVID-19 is a risk indicator of impending death in patients with less severe clinical conditions at ICU admission.

As is also known in critically ill patients without COVID-19, AKI impacts heavily on outcomes. A large meta-analysis including 110 studies of adult hospitalized patients with critical illness worldwide (21) reported a pooled mortality rate associated with AKI as high as 33%. Critically ill patients hospitalized with COVID-19 and developing AKI seem to have an even worse prognosis (4–6). In a meta-analysis by Robbins-Juarez et al. (22), the cohorts with >50% severe or critically ill patients had a pooled OR of death of 14.2 compared to patients without AKI. Together with AKI, and especially advanced stage AKI or AKI requiring KRT, age, the severity of clinical conditions and/or of organ dysfunction at ICU admission emerged as strong predictors of mortality at multivariable analyses in critically ill patients hospitalized for COVID-19 (5, 23–25). While it was shown that AKI is independently associated with increased mortality (7), no study has shown whether in critically ill patients with COVID-19 the prognostic implication of new-onset AKI bears the same significance regardless of the degree of patient severity at admission. In the study by Xia et al. (5), stage 3 AKI and higher SOFA score emerged as significant risk factors for death in the ICU. Xu et al. (25) also found a direct association between APACHE II score and 28-day mortality in critically ill patients with COVID-19, whereas Piñeiro et al. (26) observed that a SOFA score >8 at the time of AKI diagnosis was significantly associated with the need for KRT but not with in-hospital mortality in adjusted analyses. This discrepancy may suggest that the development of AKI is not uniformly related to the risk of death in patients with critical illness due to SARS-CoV-2 infection. Indeed, it is conceivable that the development of AKI *per se* may imply an important independent prognostic information only in the less severe patients, whereas it does not change prognosis in patients already at the highest risk of death. In fact, among our patients in the highest tertile of SOFA score at ICU admission, 86.5% had hemodynamic instability with the need for vasoactive support, all were mechanically ventilated, and 51.4% developed AKI (13.5% stage 3 AKI) in the ICU. Conversely, among those in the lowest SOFA tertile, only 4.1% needed vasopressors, 75.5% were mechanically ventilated, and only 16.4% developed AKI (4.1% stage 3 AKI) in the ICU. Moreover, 16.2% of the patients in the highest SOFA tertile presented with AKI at ICU admission, compared with 2.0% of those in the lowest tertile.

The trend in HRs across tertiles appeared more clear-cut with SOFA than with the APACHE II score. Indeed, the SOFA score reflects more organ dysfunction than the overall severity of clinical conditions, whereas the opposite is true for the APACHE II score. Accordingly, compared with the SOFA score, the APACHE II score encompasses a greater number of clinical and laboratory parameters, including body temperature, heart rate, respiratory rate, white blood cell count, and serum sodium and potassium concentrations, whereas it does not include serum bilirubin concentration. Thus, because of a lower number of included parameters, the SOFA score is proportionately more affected by kidney dysfunction than the APACHE II score. Since all patients in our series were critically ill, the APACHE II score was in fact remarkably high both in the patients who did and in those who did not develop AKI during ICU stay [19.1 (SD 6.8) vs. 18.6 (SD 6.8), respectively, *P* = 0.70; Supplementary Table 1]. Nevertheless, it should be appreciated that the APACHE II score also increased across tertiles of the SOFA score (Table 1), thus reflecting the increasing severity of clinical conditions in patients with increasing severity of organ dysfunction.

The fact that, in critically ill patients with COVID-19, new-onset AKI is associated with the risk of death in the ICU only in the subgroup with less severe conditions at admission is not irrelevant, as it represents a powerful and easy to detect prognostic risk indicator in these patients. Intriguingly, in another report, the patients who died earlier in the ICU also showed the fastest decline in kidney function values during ICU stay (5).

With the possible exception of hyponatremia, we did not detect a clear-cut trend toward an increasing frequency of any electrolyte disorder across SOFA tertiles in our population. Also, we failed to detect a significant association of any given electrolyte disorder with the hazard of death in this study, nor did we detect a significant interaction with general scores reflecting the severity of clinical conditions or organ dysfunction.

Electrolyte disorders were reported as being common in early studies performed in patients hospitalized for COVID-19 (12, 27–31), with hyponatremia, hypokalemia, and hypocalcemia being the most frequently encountered abnormalities. The occurrence of electrolyte disorders in these patients is associated with an increased risk of severe disease and unfavorable outcomes (32). Similar to other clinical settings, a negative prognostic impact of hyponatremia has been reported in patients hospitalized for COVID-19 with respect to ICU admission and death (15, 16). Tezcan et al. (15) found that hyponatremia remained an independent predictor of poor outcomes, including mortality, after adjustment for disease severity in non-critically ill COVID-19 patients. In fact, hyponatremia clustered with older age, worse respiratory indexes, and a higher burden of comorbidities in this investigation, thus representing rather a marker of disease severity than a true prognostic factor. In this study all patients were critically ill, and we did not detect significant differences in mean age or burden of comorbidities across tertiles of SOFA score. Thus, it is not unexpected that the severity of organ dysfunction may have obscured a putative role of hyponatremia as a prognostic marker.

On the other hand, we found a significant decreasing trend in plasma bicarbonate and plasma pH across tertiles of SOFA score. Indeed, most cases of metabolic acidosis clustered among patients in the highest SOFA tertile. Thus, not unexpectedly we detected a strong and significant interaction between metabolic acidosis and the severity of organ dysfunction with respect to the hazard of death in our patient cohort, with acidosis representing more an index of the severity of clinical conditions rather than a true prognostic indicator. In a recent retrospective observational study performed in 56 patients with COVID-19 admitted to the ICU, a higher arterial blood pH was associated with survival (13). However, by applying latent class analysis to data from a multicenter cohort study that enrolled critically ill patients with COVID-19 across 67 hospitals in the United States, Vasquez et al. (14) identified four patient phenotypes and found that those belonging to phenotype 1 were characterized by acidemia, high serum lactate, severe organ dysfunction, and the highest risk of death. Thus, acidemia seems to cluster with other markers of severe disease and organ dysfunction rather than bear an independent prognostic role in critically ill patients with COVID-19.

This study has limitations. First, it is a single-center study with relatively small sample size. However, the effect size is sufficiently large to be clearly detected. In fact, per study protocol, the patients were collected in a teaching hospital during the first pandemic wave in 2020, at which time the number of beds in ICUs across all Italian metropolitan areas were still relatively few compared with the large number of patients affected by severe SARS-CoV-2-related interstitial pneumonia. Second, our results may refer to a selected patient population, and thus may not be fully generalizable. They should be considered hypothesis-generating, and need to be confirmed in further prospective studies. However, the patients were enrolled in a tertiary care university hospital and were reasonably similar to those admitted to other large academic centers in northern Italy. Thirdly, data on previous treatment with angiotensin-converting inhibitors (ACE-i) or angiotensin receptor blockers (ARBs) were missing. As it is still debated whether the treatment with ACE-i or ARBs may impact on patient outcomes (33), we could not investigate a putative moderator effect of baseline treatment with these drugs on the prognostic impact of AKI. While we cannot exclude that previous treatment with renin-angiotensin-aldosterone system inhibitors (RAASi) may have favored AKI occurrence prior to ICU admission in patients hospitalized for severe COVID-19, because antihypertensive treatment was withdrawn in all critically ill patients at the time of ICU admission we doubt that previous treatment with RAASi may have affected the prognostic impact of new-onset AKI occurring during ICU stay. Lastly, because urine output data were not available, changes in serum creatinine were used as the unique criteria for AKI diagnosis in this study.

This study has also strengths. Serum creatinine and electrolyte values and acid–base parameters were collected on a daily basis for 28 days since the time of ICU admission in all patients, which allowed us to build a robust 28-day death risk model with time-varying predictors. Moreover, by analyzing the interaction between each of these time-varying predictors and the degree of organ dysfunction at ICU admission, we could identify a clinically significant incremental prognostic role of AKI in the patients with less severe organ dysfunction. To our knowledge, this approach has not been attempted before and may help in the early recognition of patients at high risk of death in the ICU notwithstanding a theoretically low risk based on the SOFA score at ICU admission.

## Conclusion

In a cohort of critically ill patients admitted to the ICU for severe COVID-19, we found that the development of AKI during ICU stay is a strong indicator of increased hazard of death. Conversely, metabolic acidosis represents more a marker of disease severity rather than a true prognostic risk indicator. While our results may be considered hypothesis-generating, we believe that our investigational approach deserves replication in larger cohorts of critically ill patients with, and possibly without, COVID-19.

## Data Availability Statement

The raw data supporting the conclusions of this article will be made available by the authors, without undue reservation.

## Ethics Statement

The studies involving human participants were reviewed and approved by the Comitato Etico dell’Area Vasta Emilia Nord. The ethics committee waived the requirement of written informed consent for participation.

## Author Contributions

GR and UM designed the study, analyzed the data, and wrote the manuscript draft. FDM and GDB prepared and checked the dataset. MG, MB, and EP enrolled the patients and collected the data. IG, VP, and SM provided substantial revision. EF supervised the study and revised the final version of the manuscript. All authors contributed to the article and approved the submitted version.

## Conflict of Interest

The authors declare that the research was conducted in the absence of any commercial or financial relationships that could be construed as a potential conflict of interest.

## Publisher’s Note

All claims expressed in this article are solely those of the authors and do not necessarily represent those of their affiliated organizations, or those of the publisher, the editors and the reviewers. Any product that may be evaluated in this article, or claim that may be made by its manufacturer, is not guaranteed or endorsed by the publisher.
