# Supplementary material for: The Association of New-Onset Acute Kidney Injury and Mortality in Critically Ill Patients With COVID-19 With Less Severe Clinical Conditions at Admission: A Moderation Analysis
Source: Front Med (Lausanne). 2022 Mar 18;9:799298. doi: 10.3389/fmed.2022.799298 (PMC8971281; doi:10.3389/fmed.2022.799298)
Supplement: Supplementary file 1 [file Table_1.docx]

|  | All Patients | | | Developed AKI during ICU stay | | | | | | P value* |
| --- | --- | --- | --- | --- | --- | --- | --- | --- | --- | --- |
|  |  |  |  | No | | | Yes | | |  |
| Number of patients | 115 |  |  | 81 |  |  | 34 |  |  |  |
| Age, years | 115 | 59.7 | (8.9) | 81 | 59.5 | (9.1) | 34 | 60.1 | (8.8) | 0.690 |
| Sex |  |  |  |  |  |  |  |  |  |  |
| Males |  | 21 | 18.3% |  | 19 | 23.5% |  | 2 | 5.9% | 0.033 |
| Females |  | 94 | 81.7% |  | 62 | 76.5% |  | 32 | 94.1% |  |
| APACHE II score | 115 | 18.7 | (6.8) | 81 | 18.6 | (6.8) | 34 | 19.1 | (6.8) | 0.699 |
| SOFA score | 115 | 6.2 | (2.6) | 81 | 5.8 | (2.6) | 34 | 7.2 | (2.4) | 0.004 |
| Charlson's score - Age | 115 | 2.0 | (1.2) | 81 | 2.1 | (1.3) | 34 | 1.9 | (1.0) | 0.905 |
| Obesity |  |  |  |  |  |  |  |  |  |  |
| No |  | 98 | 85.2% |  | 69 | 85.2% |  | 29 | 85.3% | 1.000 |
| Yes |  | 17 | 14.8% |  | 12 | 14.8% |  | 5 | 14.7% |  |
| Hypertension | 115 |  |  | 81 |  |  | 34 |  |  |  |
| No |  | 60 | 52.2% |  | 38 | 46.9% |  | 22 | 64.7% | 0.103 |
| Yes |  | 55 | 47.8% |  | 43 | 53.1% |  | 12 | 35.3% |  |
| Diabetes | 115 |  |  | 81 |  |  | 34 |  |  |  |
| No |  | 100 | 87.0% |  | 72 | 88.9% |  | 28 | 82.4% | 0.371 |
| Yes |  | 15 | 13.0% |  | 9 | 11.1% |  | 6 | 17.6% |  |
| CKD | 115 |  |  | 81 |  |  | 34 |  |  |  |
| No |  | 109 | 94.8% |  | 77 | 95.1% |  | 32 | 94.1% | 1.000 |
| Yes |  | 6 | 5.2% |  | 4 | 4.9% |  | 2 | 5.9% |  |
| CAD | 115 |  |  | 81 |  |  | 34 |  |  |  |
| No |  | 108 | 93.9% |  | 76 | 93.8% |  | 32 | 94.1% | 1.000 |
| Yes |  | 7 | 6.1% |  | 5 | 6.2% |  | 2 | 5.9% |  |
| COPD | 115 |  |  | 81 |  |  | 34 |  |  |  |
| No |  | 111 | 96.5% |  | 77 | 95.1% |  | 34 | 100.0% | 0.317 |
| Yes |  | 4 | 3.5% |  | 4 | 4.9% |  | 0 | 0.0% |  |
| Cancer | 115 |  |  | 81 |  |  | 34 |  |  |  |
| No |  | 110 | 95.7% |  | 77 | 95.1% |  | 33 | 97.1% | 1.000 |
| Yes |  | 5 | 4.3% |  | 4 | 4.9% |  | 1 | 2.9% |  |
| Sepsis | 115 |  |  | 81 |  |  | 34 |  |  |  |
| No |  | 92 | 80.0% |  | 68 | 84.0% |  | 24 | 70.6% | 0.127 |
| Yes |  | 23 | 20.0% |  | 13 | 16.0% |  | 10 | 29.4% |  |
| MBP, mmHg | 114 | 79.5 | (11.6) | 81 | 80.7 | (12.0) | 33 | 76.5 | (10.0) | 0.056 |
| HR, bpm | 114 | 83.2 | (20.3) | 81 | 81.4 | (19.2) | 33 | 87.5 | (22.5) | 0.118 |
| Respiratory rate, breaths/min | 115 | 20.0 | (4.5) | 81 | 19.8 | (4.5) | 34 | 20.3 | (4.5) | 0.586 |
| Body temperature, °C | 114 | 36.7 | (1.0) | 81 | 36.6 | (1.0) | 33 | 36.9 | (1.1) | 0.187 |
| Fever | 115 |  |  | 81 |  |  | 34 |  |  |  |
| No |  | 9 | 7.8% |  | 6 | 7.4% |  | 3 | 8.8% | 0.723 |
| Yes |  | 106 | 92.2% |  | 75 | 92.6% |  | 31 | 91.2% |  |
| Dyspnea | 115 |  |  | 81 |  |  | 34 |  |  |  |
| No |  | 45 | 39.1% |  | 33 | 40.7% |  | 12 | 35.3% | 0.677 |
| Yes |  | 70 | 60.9% |  | 48 | 59.3% |  | 22 | 64.7% |  |
| Cough | 115 |  |  | 81 |  |  | 34 |  |  |  |
| No |  | 59 | 51.3% |  | 36 | 44.4% |  | 23 | 67.6% | 0.026 |
| Yes |  | 56 | 48.7% |  | 45 | 55.6% |  | 11 | 32.4% |  |
| Diarrhea | 115 |  |  | 81 |  |  | 34 |  |  |  |
| No |  | 107 | 93.0% |  | 75 | 92.6% |  | 32 | 94.1% | 1.000 |
| Yes |  | 8 | 7.0% |  | 6 | 7.4% |  | 2 | 5.9% |  |
| Oliguria | 48 |  |  | 15 |  |  | 33 |  |  |  |
| No |  | 40 | 83.3% |  | 14 | 93.3% |  | 26 | 78.8% | 0.406 |
| Yes |  | 8 | 16.7% |  | 1 | 6.7% |  | 7 | 21.2% |  |
| Time from symptom onset to ICU admission, days | 114 | 12.6 | (7.7) | 81 | 13.2 | (8.5) | 33 | 11.2 | (5.1) | 0.220 |
| Fluid balance in the first 24 hours, mL | 110 | 1364.0 | (1245.5) | 78 | 1241.6 | (1200.6) | 32 | 1662.4 | (1320.8) | 0.183 |
| Vasopressors | 115 |  |  | 81 |  |  | 34 |  |  |  |
| No |  | 64 | 55.7% |  | 52 | 64.2% |  | 12 | 35.3% | 0.007 |
| Yes |  | 51 | 44.3% |  | 29 | 35.8% |  | 22 | 64.7% |  |
| MV | 115 |  |  | 81 |  |  | 34 |  |  |  |
| No |  | 13 | 11.3% |  | 12 | 14.8% |  | 1 | 2.9% | 0.104 |
| Yes |  | 102 | 88.7% |  | 69 | 85.2% |  | 33 | 97.1% |  |
| WBC count, x103/?L | 115 | 10735.3 | (5518.2) | 81 | 10530.7 | (5697.9) | 34 | 11222.7 | (5112.9) | 0.313 |
| Hemoglobin, g/dL | 115 | 12.5 | (1.7) | 81 | 12.5 | (1.7) | 34 | 12.4 | (1.6) | 0.839 |
| MCV, fL | 115 | 89.4 | (12.3) | 81 | 88.4 | (14.2) | 34 | 91.7 | (5.3) | 0.261 |
| PLT count, x103/?L | 115 | 260217.4 | (100229.5) | 81 | 256814.8 | (94482.3) | 34 | 268323.5 | (113873.3) | 0.948 |
| Serum glucose, mg/dL | 106 | 156.7 | (61.1) | 74 | 156.2 | (62.7) | 32 | 157.7 | (58.1) | 0.633 |
| Serum urea, mg/dL | 113 | 56.3 | (45.6) | 79 | 54.4 | (49.0) | 34 | 60.8 | (36.9) | 0.061 |
| Serum creatinine, mg/dL | 115 | 0.9 | (0.7) | 81 | 0.8 | (0.7) | 34 | 1.1 | (0.7) | 0.000 |
| Serum sodium, mmol/L | 115 | 137.5 | (4.0) | 81 | 136.9 | (3.7) | 34 | 139.0 | (4.3) | 0.024 |
| Serum potassium, mmol/L | 115 | 4.0 | (0.5) | 81 | 4.0 | (0.6) | 34 | 3.9 | (0.5) | 0.777 |
| Serum chloride, mmol/L | 112 | 101.5 | (4.6) | 78 | 100.7 | (4.5) | 34 | 103.5 | (4.3) | 0.002 |
| Serum calcium, mg/dL | 94 | 8.0 | (0.7) | 66 | 8.1 | (0.7) | 28 | 7.8 | (0.4) | 0.065 |
| Serum total bilirubin, mg/dL | 107 | 0.9 | (0.5) | 75 | 0.9 | (0.6) | 32 | 0.9 | (0.4) | 0.623 |
| AST, UI/L | 109 | 79.7 | (99.5) | 76 | 76.3 | (68.1) | 33 | 87.5 | (149.8) | 0.933 |
| ALT, UI/L | 107 | 67.0 | (75.6) | 74 | 73.6 | (83.8) | 33 | 52.2 | (51.0) | 0.136 |
| LDH, UI/L | 96 | 602.7 | (218.3) | 68 | 575.7 | (213.7) | 28 | 668.3 | (219.1) | 0.044 |
| CPK, UI/L | 96 | 313.3 | (820.4) | 65 | 335.0 | (990.9) | 31 | 267.8 | (183.8) | 0.018 |
| INR | 109 | 1.4 | (0.3) | 78 | 1.4 | (0.2) | 31 | 1.5 | (0.3) | 0.028 |
| aPTT ratio | 111 | 1.0 | (0.3) | 79 | 1.0 | (0.3) | 32 | 1.0 | (0.1) | 0.991 |
| D-Dimer, ng/mL | 102 | 4384.1 | (3395.6) | 73 | 3903.5 | (3156.0) | 29 | 5593.9 | (3723.0) | 0.037 |
| Serum CRP, mg/L | 88 | 160.0 | (81.7) | 62 | 144.3 | (84.1) | 26 | 197.5 | (62.5) | 0.005 |
| Serum PCT, ng/mL | 113 | 4.2 | (30.3) | 80 | 4.7 | (35.9) | 33 | 3.0 | (5.2) | 0.000 |
| Serum troponine, ng/L | 98 | 43.9 | (76.1) | 68 | 40.3 | (79.1) | 30 | 52.1 | (69.2) | 0.032 |
| Arterial blood pH | 115 | 7.4 | (0.1) | 81 | 7.4 | (0.1) | 34 | 7.3 | (0.1) | 0.010 |
| Partial pressure of O2 in arterial blood, mmHg | 115 | 80.2 | (29.5) | 81 | 81.9 | (30.9) | 34 | 76.3 | (25.7) | 0.390 |
| Partial pressure of CO2 in arterial blood, mmHg | 115 | 46.5 | (11.1) | 81 | 45.2 | (10.0) | 34 | 49.5 | (13.0) | 0.169 |
| Bicarbonate concentration in arterial blood, mmol/L | 115 | 24.5 | (4.0) | 81 | 25.0 | (3.7) | 34 | 23.2 | (4.4) | 0.003 |
| eGFR, mL/min/1.73 m2 | 56 | 85.6 | (13.1) | 38 | 84.7 | (14.7) | 18 | 87.5 | (8.7) | 0.605 |
| Hypernatremia | 115 |  |  | 81 |  |  | 34 |  |  |  |
| No |  | 110 | 95.7% |  | 79 | 97.5% |  | 31 | 91.2% | 0.153 |
| Yes |  | 5 | 4.3% |  | 2 | 2.5% |  | 3 | 8.8% |  |
| Hyponatremia | 115 |  |  | 81 |  |  | 34 |  |  |  |
| No |  | 94 | 81.7% |  | 65 | 80.2% |  | 29 | 85.3% | 0.606 |
| Yes |  | 21 | 18.3% |  | 16 | 19.8% |  | 5 | 14.7% |  |
| Hyperkalemia | 115 |  |  | 81 |  |  | 34 |  |  |  |
| No |  | 114 | 99.1% |  | 80 | 98.8% |  | 34 | 100.0% | 1.000 |
| Yes |  | 1 | 0.9% |  | 1 | 1.2% |  | 0 | 0.0% |  |
| Hypokalemia | 115 |  |  | 81 |  |  | 34 |  |  |  |
| No |  | 97 | 84.3% |  | 67 | 82.7% |  | 30 | 88.2% | 0.580 |
| Yes |  | 18 | 15.7% |  | 14 | 17.3% |  | 4 | 11.8% |  |
| Metabolic alkalosis | 115 |  |  | 81 |  |  | 34 |  |  |  |
| No |  | 95 | 82.6% |  | 63 | 77.8% |  | 32 | 94.1% | 0.056 |
| Yes |  | 20 | 17.4% |  | 18 | 22.2% |  | 2 | 5.9% |  |
| Metabolic acidosis | 115 |  |  | 81 |  |  | 34 |  |  |  |
| No |  | 89 | 77.4% |  | 68 | 84.0% |  | 21 | 61.8% | 0.014 |
| Yes |  | 26 | 22.6% |  | 13 | 16.0% |  | 13 | 38.2% |  |
| Treatment with hydroxycloroquine | 115 |  |  | 81 |  |  | 34 |  |  |  |
| No |  | 8 | 7.0% |  | 5 | 6.2% |  | 3 | 8.8% | 0.692 |
| Yes |  | 107 | 93.0% |  | 76 | 93.8% |  | 31 | 91.2% |  |
| Treatment with antiviral drugs | 115 |  |  | 81 |  |  | 34 |  |  |  |
| No |  | 4 | 3.5% |  | 4 | 4.9% |  | 0 | 0.0% | 0.317 |
| Yes |  | 111 | 96.5% |  | 77 | 95.1% |  | 34 | 100.0% |  |
| Treatment with azithromycin | 115 |  |  | 81 |  |  | 34 |  |  |  |
| No |  | 30 | 26.1% |  | 15 | 18.5% |  | 15 | 44.1% | 0.009 |
| Yes |  | 85 | 73.9% |  | 66 | 81.5% |  | 19 | 55.9% |  |
| Treatment with tocilizumab | 115 |  |  | 81 |  |  | 34 |  |  |  |
| No |  | 101 | 87.8% |  | 69 | 85.2% |  | 32 | 94.1% | 0.226 |
| Yes |  | 14 | 12.2% |  | 12 | 14.8% |  | 2 | 5.9% |  |
| Treatment with colchicine | 115 |  |  | 81 |  |  | 34 |  |  |  |
| No |  | 107 | 93.0% |  | 75 | 92.6% |  | 32 | 94.1% | 1.000 |
| Yes |  | 8 | 7.0% |  | 6 | 7.4% |  | 2 | 5.9% |  |
| AKI at hospital admission | 115 |  |  | 81 |  |  | 34 |  |  |  |
| No |  | 98 | 85.2% |  | 68 | 84.0% |  | 30 | 88.2% | 0.774 |
| Yes |  | 17 | 14.8% |  | 13 | 16.0% |  | 4 | 11.8% |  |
| AKI at ICU admission | 115 |  |  | 81 |  |  | 34 |  |  |  |
| No |  | 107 | 93.0% |  | 77 | 95.1% |  | 30 | 88.2% | 0.233 |
| Yes |  | 8 | 7.0% |  | 4 | 4.9% |  | 4 | 11.8% |  |
| Days with NIV or MV | 115 | 28.4 | (27.2) | 81 | 31.0 | (29.9) | 34 | 22.1 | (18.2) | 0.421 |
| Lenght of ICU stay, days | 115 | 30.4 | (29.8) | 81 | 33.8 | (32.7) | 34 | 22.3 | (19.5) | 0.190 |

Data are expressed as mean (SD), or count and percentage. The number of patients in the whole patient population and in the subgroups of patients who did and those who did not develop AKI during ICU stay are reported for each variable in the second, fifth and eighth column, respectively

*P values refer to comparison between the patients who did and thos who did not develop AKI during ICU stay by the t-test for continuous variables and the Fisher exact test for dichotomous variables).

ALT, alanine aminotransferase; APACHE II, Acute Physiology Assessment and Chronic Health Evaluation II; aPTT, activated partial thromboplastin time; AST, aspartate aminotransferase; CAD, coronary artery disease; CKD, chronic kidney disease; COPD, chronic obstructive pulmonary disease; CPK, creatine phosphokinase; CRP, C-reactive protein; eGFR, estimated glomerular filtration rate; ICU, intensive care unit; INR, international normalized ratio; MBP, mean blood pressure; MCV, mean corpuscular volume; MV, mechanical ventilation; NIV, noninvasive ventilation; PCT, procalcitonin; PLT, platelet; SOFA, Sequential Organ Failure Assessment; WBC, white blood cell.
