# Supplementary material for: The Association of New-Onset Acute Kidney Injury and Mortality in Critically Ill Patients With COVID-19 With Less Severe Clinical Conditions at Admission: A Moderation Analysis
Source: Front Med (Lausanne). 2022 Mar 18;9:799298. doi: 10.3389/fmed.2022.799298 (PMC8971281; doi:10.3389/fmed.2022.799298)
Supplement: Supplementary file 2 [file Table_2.docx]

|  | SOFA tertiles | | | |
| --- | --- | --- | --- | --- |
|  | <=5 | >5 & <=7 | >7 | Total |
|  |  |  |  |  |
| Age, years | 49 | 29 | 37 | 115 |
|  | 31.0 | 49.0 | 46.0 | 31.0 |
|  | 75.0 | 74.0 | 80.0 | 80.0 |
| APACHE II score | 49 | 29 | 37 | 115 |
|  | 6.0 | 11.0 | 14.0 | 6.0 |
|  | 22.0 | 30.0 | 37.0 | 37.0 |
| SOFA score | 49 | 29 | 37 | 115 |
|  | 0.0 | 6.0 | 8.0 | 0.0 |
|  | 5.0 | 7.0 | 15.0 | 15.0 |
| Charlson's score - Age | 49 | 29 | 37 | 115 |
|  | 0.0 | 0.0 | 0.0 | 0.0 |
|  | 5.0 | 5.0 | 5.0 | 5.0 |
| MBP, mmHg | 49 | 29 | 36 | 114 |
|  | 64.0 | 65.0 | 60.0 | 60.0 |
|  | 110.0 | 115.0 | 100.0 | 115.0 |
| HR, bpm | 49 | 29 | 36 | 114 |
|  | 50.0 | 53.0 | 55.0 | 50.0 |
|  | 115.0 | 150.0 | 150.0 | 150.0 |
| Respiratory rate, breaths/min | 49 | 29 | 37 | 115 |
|  | 8.0 | 16.0 | 12.0 | 8.0 |
|  | 42.0 | 35.0 | 32.0 | 42.0 |
| Body temperature, °C | 49 | 29 | 36 | 114 |
|  | 34.0 | 35.0 | 34.4 | 34.0 |
|  | 39.0 | 38.8 | 38.9 | 39.0 |
| Time from symptom onset to ICU admission, days | 49 | 29 | 36 | 114 |
|  | 5.0 | 4.7 | -24.8 | -24.8 |
|  | 46.8 | 39.9 | 32.9 | 46.8 |
| Fluid balance in the first 24 hours, mL | 47 | 28 | 35 | 110 |
|  | -1104.0 | -200.0 | -1140.0 | -1140.0 |
|  | 4544.0 | 2756.0 | 4900.0 | 4900.0 |
| WBC count, x103/?L | 49 | 29 | 37 | 115 |
|  | 4000.0 | 4290.0 | 3320.0 | 3320.0 |
|  | 20040.0 | 32880.0 | 24750.0 | 32880.0 |
| Hemoglobin, g/dL | 49 | 29 | 37 | 115 |
|  | 10.2 | 7.8 | 8.7 | 7.8 |
|  | 16.9 | 15.8 | 16.6 | 16.9 |
| MCV, fL | 49 | 29 | 37 | 115 |
|  | 9.4 | 62.4 | 5.4 | 5.4 |
|  | 98.5 | 99.2 | 106.8 | 106.8 |
| PLT count, x103/?L | 49 | 29 | 37 | 115 |
|  | 132000.0 | 107000.0 | 93000.0 | 93000.0 |
|  | 522000.0 | 488000.0 | 606000.0 | 606000.0 |
| Serum glucose, mg/dL | 45 | 26 | 35 | 106 |
|  | 73.0 | 77.0 | 91.0 | 73.0 |
|  | 327.0 | 353.0 | 435.0 | 435.0 |
| Serum urea, mg/dL | 48 | 28 | 37 | 113 |
|  | 19.0 | 23.0 | 18.0 | 18.0 |
|  | 85.0 | 242.0 | 370.0 | 370.0 |
| Serum creatinine, mg/dL | 49 | 29 | 37 | 115 |
|  | 0.3 | 0.4 | 0.4 | 0.3 |
|  | 1.1 | 2.6 | 6.1 | 6.1 |
| Serum sodium, mmol/L | 49 | 29 | 37 | 115 |
|  | 129.0 | 128.0 | 130.0 | 128.0 |
|  | 145.0 | 146.0 | 148.0 | 148.0 |
| Serum potassium, mmol/L | 49 | 29 | 37 | 115 |
|  | 2.7 | 3.0 | 3.1 | 2.7 |
|  | 5.0 | 5.5 | 5.5 | 5.5 |
| Serum chloride, mmol/L | 48 | 27 | 37 | 112 |
|  | 90.0 | 91.0 | 91.0 | 90.0 |
|  | 107.0 | 110.0 | 111.0 | 111.0 |
| Serum calcium, mg/dL | 40 | 22 | 32 | 94 |
|  | 7.2 | 5.7 | 7.1 | 5.7 |
|  | 10.5 | 9.6 | 9.7 | 10.5 |
| Serum total bilirubin, mg/dL | 46 | 26 | 35 | 107 |
|  | 0.4 | 0.3 | 0.3 | 0.3 |
|  | 1.6 | 4.0 | 2.9 | 4.0 |
| AST, UI/L | 47 | 26 | 36 | 109 |
|  | 21.0 | 22.0 | 21.0 | 21.0 |
|  | 410.0 | 270.0 | 907.0 | 907.0 |
| ALT, UI/L | 47 | 25 | 35 | 107 |
|  | 13.0 | 12.0 | 8.0 | 8.0 |
|  | 405.0 | 490.0 | 301.0 | 490.0 |
| LDH, UI/L | 43 | 24 | 29 | 96 |
|  | 83.4 | 272.0 | 296.0 | 83.4 |
|  | 1137.0 | 1094.0 | 1170.0 | 1170.0 |
| CPK, UI/L | 41 | 24 | 31 | 96 |
|  | 25.0 | 45.0 | 23.0 | 23.0 |
|  | 1505.0 | 1164.0 | 7883.0 | 7883.0 |
| INR | 45 | 29 | 35 | 109 |
|  | 1.0 | 1.1 | 1.1 | 1.0 |
|  | 2.4 | 2.0 | 2.2 | 2.4 |
| aPTT ratio | 46 | 29 | 36 | 111 |
|  | 0.8 | 0.8 | 0.7 | 0.7 |
|  | 1.6 | 2.7 | 2.9 | 2.9 |
| D-Dimer, ng/mL | 47 | 25 | 30 | 102 |
|  | 480.0 | 434.0 | 416.0 | 416.0 |
|  | 9000.0 | 9000.0 | 9000.0 | 9000.0 |
| Serum CRP, mg/L | 43 | 20 | 25 | 88 |
|  | 4.1 | 0.5 | 4.9 | 0.5 |
|  | 250.0 | 250.0 | 250.0 | 250.0 |
| Serum PCT, ng/mL | 49 | 29 | 35 | 113 |
|  | 0.0 | 0.1 | 0.0 | 0.0 |
|  | 28.0 | 7.4 | 322.0 | 322.0 |
| Serum troponin, ng/L | 39 | 24 | 35 | 98 |
|  | 2.3 | 3.9 | 4.9 | 2.3 |
|  | 212.5 | 544.7 | 286.6 | 544.7 |
| Arterial blood pH | 49 | 29 | 37 | 115 |
|  | 7.2 | 7.1 | 6.9 | 6.9 |
|  | 7.5 | 7.5 | 7.5 | 7.5 |
| Partial pressure of O2 in arterial blood, mmHg | 49 | 29 | 37 | 115 |
|  | 39.0 | 32.9 | 38.0 | 32.9 |
|  | 197.0 | 158.0 | 185.0 | 197.0 |
| Partial pressure of CO2 in arterial blood, mmHg | 49 | 29 | 37 | 115 |
|  | 28.0 | 28.0 | 32.0 | 28.0 |
|  | 73.0 | 81.0 | 85.0 | 85.0 |
| Bicarbonate concentration in arterial blood, mmol/L | 49 | 29 | 37 | 115 |
|  | 19.1 | 14.6 | 17.2 | 14.6 |
|  | 33.0 | 39.8 | 41.6 | 41.6 |
| eGFR, mL/min/1.73 m2 | 26 | 17 | 13 | 56 |
|  | 68.0 | 47.0 | 67.0 | 47.0 |
|  | 109.0 | 100.0 | 101.0 | 109.0 |
| Days with MV | 49 | 29 | 37 | 115 |
|  | 0.0 | 2.0 | 2.0 | 0.0 |
|  | 92.0 | 104.0 | 122.0 | 122.0 |
| Lenght of ICU stay, days | 49 | 29 | 37 | 115 |
|  | 1.6 | 1.2 | 1.1 | 1.1 |
|  | 108.4 | 108.0 | 127.8 | 127.8 |
| Death at 28 days since ICU admission |  |  |  |  |
| No | 35 | 13 | 21 | 69 |
|  | 71.43 | 44.83 | 56.76 | 60.00 |
| Yes | 14 | 16 | 16 | 46 |
|  | 28.57 | 55.17 | 43.24 | 40.00 |

The first row in each variable denotes the number of patients in each SOFA score tertile and in the whole population. The second and the third row denote minimum and maximum values for each continuous variable in each SOFA score tertile and in the whole patient population.

ALT, alanine aminotransferase; APACHE II, Acute Physiology and Chronic Health Evaluation II; aPTT, activated partial thromboplastin time; AST, aspartate aminotransferase; CPK, creatine phosphokinase; CRP, C-reactive protein; eGFR, estimated glomerular filtration rate; ICU, intensive care unit; INR, international normalized ratio; MCV, mean corpuscular volume; MV, mechanical ventilation; PCT, procalcitonin; PLT, platelet; SOFA, Sequential Organ Failure Assessment; WBC, white blood cell.
