# Supplementary material for: The Association of New-Onset Acute Kidney Injury and Mortality in Critically Ill Patients With COVID-19 With Less Severe Clinical Conditions at Admission: A Moderation Analysis
Source: Front Med (Lausanne). 2022 Mar 18;9:799298. doi: 10.3389/fmed.2022.799298 (PMC8971281; doi:10.3389/fmed.2022.799298)
Supplement: Supplementary file 3 [file Table_3.docx]

| **Variables** |  | **Crude** | **SOFA-adjusted-Model** | **Fully-adjusted-Model** |
| --- | --- | --- | --- | --- |
|  | HR | 2.43*** | 1.86* | 1.90* |
| AKI during ICU stay | 95% CI | [1.36,4.32] | [0.95,3.65] | [0.96,3.75] |
|  | P | 0.003 | 0.072 | 0.066 |
|  |  |  |  |  |
|  | HR | 1.14** | 1.12* | 1.12 |
| SOFA score | 95% CI | [1.01,1.28] | [0.99,1.26] | [0.97,1.28] |
|  | P | 0.027 | 0.072 | 0.116 |
|  |  |  |  |  |
|  | HR | 1.35 |  | 1.15 |
| AKI at admission | 95% CI | [0.58,3.16] |  | [0.42,3.14] |
|  | P | 0.484 |  | 0.786 |
|  |  |  |  |  |
|  | HR | 1.07*** |  | 1.07** |
| Age, years | 95% CI | [1.03,1.11] |  | [1.01,1.13] |
|  | P | 0.000 |  | 0.013 |
|  |  |  |  |  |
|  | HR | 1.39*** |  | 0.95 |
| Charlson's' Index (-age component) | 95% CI | [1.13,1.72] |  | [0.65,1.39] |
|  | P | 0.002 |  | 0.788 |
|  |  |  |  |  |
|  | HR | 0.77 |  | 0.70 |
| Male sex | 95% CI | [0.42,1.42] |  | [0.33,1.51] |
|  | P | 0.407 |  | 0.367 |
|  |  |  |  |  |
|  |  |  |  |  |
| **Variables** |  | **Crude** | **APACHE II-adjusted-Model** | **Fully-adjusted-Model** |
|  | HR | 2.43*** | 2.10* | 2.15* |
| AKI during ICU stay | 95% CI | [1.36,4.32] | [1.09,4.07] | [1.10,4.20] |
|  | P | 0.003 | 0.027 | 0.025 |
|  |  |  |  |  |
|  | HR | 1.06*** | 1.06*** | 1.03 |
| APACHE II score | 95% CI | [1.02,1.10] | [1.02,1.10] | [0.99,1.09] |
|  | P | 0.007 | 0.008 | 0.173 |
|  |  |  |  |  |
|  | HR | 1.35 |  | 1.15 |
| AKI at admission | 95% CI | [0.58,3.16] |  | [0.40,3.25] |
|  | P | 0.484 |  | 0.797 |
|  |  |  |  |  |
|  | HR | 1.07*** |  | 1.06** |
| Age, years | 95% CI | [1.03,1.11] |  | [1.00,1.12] |
|  | P | 0.000 |  | 0.045 |
|  |  |  |  |  |
|  | HR | 1.39*** |  | 0.97 |
| Charlson's' Index (-age component) | 95% CI | [1.13,1.72] |  | [0.67,1.42] |
|  | P | 0.002 |  | 0.888 |
|  |  |  |  |  |
|  | HR | 0.77 |  | 0.77 |
| Male sex | 95% CI | [0.42,1.42] |  | [0.37,1.63] |
|  | P | 0.407 |  | 0.495 |

*P<0.10

**P<0.05

***P<0.01

AKI, acute kidney injury; APACHE II, Acute Physiology and Chronic Health Evluation II; CI, confidence interval; HR, hazard ratio; ICU, intensive care unit; SOFA, Sequential Organ Failure Asessment
